# Delayed arachnoid ossification following lumbar decompression and fusion surgery: A case report and review of literature

**DOI:** 10.1097/MD.0000000000046544

**Published:** 2025-12-19

**Authors:** Xin Xin, Ze-Lin Yue, Tong-Hao Sheng, Chuang-Ye Zhang, Jing-Guo Wu, Chuan-Fu Wei, Nian-Hu Li

**Affiliations:** a First Clinical College of Medicine, Shandong University of Traditional Chinese Medicine, Jinan, China; b Department of Spine and Spinal Cord Surgery, Affiliated Hospital of Shandong University of Traditional Chinese Medicine, Jinan, China.

**Keywords:** arachnoid ossification (AO), conservative management, non-contrast CT scans, postoperative complications of lumbar spine

## Abstract

**Rationale:**

Lumbar Decompression and Fusion Surgery is an effective and safe surgical technique widely used for treating spondylolisthesis. Arachnoid ossification (AO) is a rare condition associated with neurological dysfunction after lumbar spine surgery, with limited reports in the literature. In this report, we present a rare case of AO detected 1 year after lumbar fusion, with serial imaging first revealing its emergence and guiding early detection and intervention.

**Patient concerns:**

A 61-year-old female who experienced progressive pain and numbness in both lower extremities 2 years after undergoing lumbar decompression and fusion surgery.

**Diagnoses:**

Based on the medical history, symptoms and imaging studies, the patient was diagnosed with Delayed AO.

**Interventions:**

After discussing the condition and evaluating surgical options, a conservative treatment regimen was implemented, including nonsteroidal anti-inflammatory drugs (NSAIDs) for analgesia and anti-inflammatory effects, methylcobalamin for neurotrophic support, mannitol combined with corticosteroids to reduce neuroinflammatory edema, and adjunctive acupuncture therapy.

**Outcomes:**

There was a partial relief of symptoms including pain and numbness in both lower limbs on the 7th day after systemic treatment. These symptoms had improved by the 12th day; however, mild pain and discomfort persisted in the right foot. Following symptom alleviation, the patient was discharged from the hospital.

**Lessons:**

Patients may experience pain and numbness in both lower limbs after lumbar decompression and fusion surgery. This condition is likely attributed to the compression of spinal nerves caused by AO within the spinal canal. Given the significant surgical risks associated with this procedure, a systematic conservative treatment approach is recommended, which can yield satisfactory therapeutic outcomes.

## 1. Introduction

Arachnoid ossification (AO) is a rare pathological entity that can follow infection, trauma, or chemical insult. Reports of AO after spinal surgery are relatively sparse, and neither standardized guidelines nor longitudinal data exist.^[1]^ We present a case manifesting 1 year after lumbar decompression and fusion, with serial imaging of the lumbar arachnoid space at postoperative days 3 and months 3, 6, 10, 14, and 25, and review the literature on etiology, pathogenesis, diagnosis, and treatment.

## 2. Case presentation

**Figure 1.**
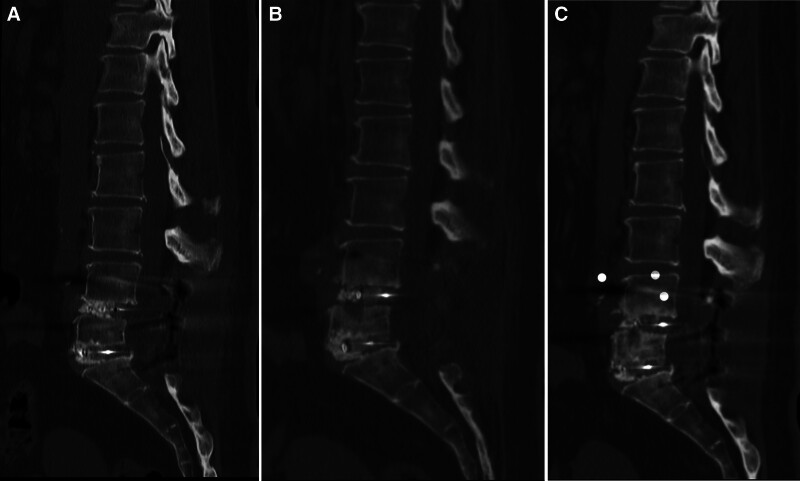
Sagittal CT imaging after surgery: (A) shows no evidence of ossification within the spinal canal on postoperative day 2. (B) shows no signs of ossification within the spinal canal on postoperative month 3. (C) continues to demonstrate no ossification within the spinal canal postoperative on month 6. CT = computed tomography.

A 61-year-old woman presented with a 1-year history of progressive bilateral lower-limb pain and numbness radiating from the gluteal regions along the posterior thighs, posterolateral calves, and into the soles. Exercise tolerance was limited by neurogenic claudication to approximately 100 m. Neurological examination revealed no overt motor weakness, and pathological reflexes were absent. She was previously healthy and denied any history of chronic illnesses such as hypertension or diabetes mellitus. Her past medical history included a thyroidectomy in 2016 for which she was taking levothyroxine. No additional surgical procedures had been performed, and the patient was not receiving any other medications. In November 2022, she underwent posterior lumbar decompression and fusion surgery for lumbar spinal stenosis which was diagnosed based on imaging findings, along with symptoms of low back pain and bilateral lower-extremity discomfort. Bilateral pedicle screws were inserted at the responsible vertebral levels. The spinous processes, laminae, and portions of the facet joints were resected to achieve thorough spinal canal decompression. After discectomy, titanium rods were used to rigidly connect the screws, and an interbody cage packed with morselized autograft was placed into the disc space (Fig. 1A). The wound was closed in anatomical layers. Her symptoms resolved postoperatively. At the 3- (Fig. 1B) and 6- (Fig. 1C) month postoperative follow-up visits, the patient reported no appreciable discomfort.

**Figure 2.**
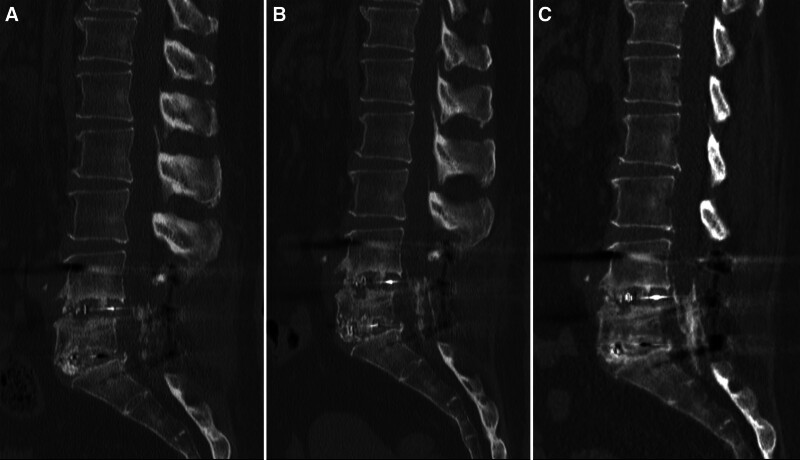
Sagittal CT images of ossification progression: (A) identifies the presence of a small amount of filamentous ossification within the spinal canal on postoperative month 10. (B) reveals an increase in filamentous ossification compared to previous scans, forming a reticular pattern on postoperative month 14. (C) shows progressive ossification, with a denser and more extensive reticular pattern on postoperative month 25. CT = computed tomography.

In September 2023, the patient developed numbness in her right foot without any apparent precipitating factors. Lumbar computed tomography (CT) revealed a small amount of reticular high-density material within the spinal canal (Fig. 2A). She was treated with topical nonsteroidal anti-inflammatory drugs (NSAIDs) and acupuncture. At the 14-month postoperative follow-up, the patient remained asymptomatic; however, CT revealed progression of the ossified lesion compared with prior imaging (Fig. 2B).

By December 2024, her symptoms worsened, with pain and numbness progressing in both lower extremities. Follow-up lumbar CT demonstrated a significant increase in reticular ossification compared to the prior imaging. The patient sought further evaluation and treatment at our institution.

On admission, the patient reported persistent bilateral lower-extremity pain and numbness that initially involved the feet and was accompanied by mild ambulatory limitation. Sensory function was normal in both lower extremities and the saddle area, and muscle tone showed normal in both lower extremities. Reduction in muscle strength of the bilateral lower limbs. No pathological reflexes were elicited.

Normal bilateral dorsalis pedis and posterior tibial artery pulses. Visual analog scale: 5; Japanese Orthopaedic Association Score: 13.

**Figure 3.**
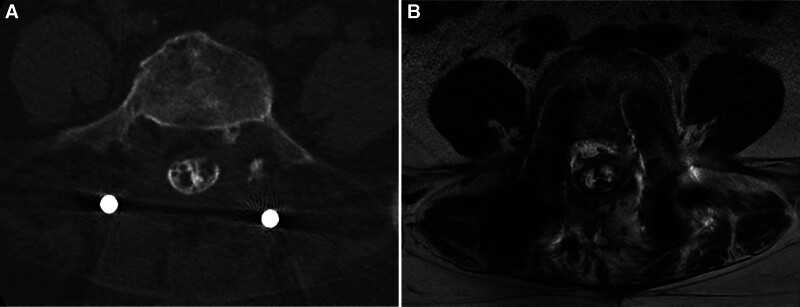
Axial CT imaging and Axial T2-weighted MRI of the ossification outcome: (A) demonstrates the CT of a characteristic honeycomb appearance of ossified areas, with calcified deposits penetrating the dural sac on postoperative month 25. (B) shows the MRI of low-signal intensity thickening in the dural sac and intradural regions, consistent with calcification and ossification on postoperative month 25. CT = computed tomography, MRI = magnetic resonance imaging.

Laboratory findings demonstrated a subnormal serum thyroid-stimulating hormone level of 0.408 μIU/mL; all remaining parameters were within their respective reference ranges. Imaging Findings: A honeycomb-like hyperintense lesion is noted within the spinal canal on imaging (Figs. 2C and 3A). Contrast-enhanced magnetic resonance imaging reveals structural disarray in the soft tissues posterior to the vertebral bodies and within the spinal canal, showing patchy areas of hyperintensity on fat-suppressed sequences (Fig. 3B). The patient was diagnosed with AO. After discussing the condition and evaluating surgical options, a conservative treatment regimen was implemented, including NSAIDs (Parecoxib Sodium Injection, 40mg/d) for analgesia and anti-inflammatory effects, methylcobalamin (Mecobalamin Injection, 1mg/d) for neurotrophic support, mannitol(Mannitol injection, 125ml/d) combined with corticosteroids(Dexamethasone Sodium Phosphate Injection, 5mg/d) to reduce neuroinflammatory edema, and adjunctive acupuncture therapy. Twelve days of conservative therapy alleviated bilateral pain and numbness (residual right-foot discomfort only), improved strength, lowered visual analog scale to 2, and raised Japanese Orthopaedic Association to 20; discharge followed. The symptoms did not recur during the 2-month follow-up after discharge and without any adverse or unanticipated events.

## 3. Discussion

In contrast to incidental leptomeningeal calcification and extradural ossification, AO is distinctly rare, as the arachnoid membrane is generally regarded as a structurally inert tissue.^[2]^ Previous studies have postulated that the pathogenesis of AO chiefly involves chronic inflammation, infection-driven processes, disturbances in calcium metabolism, and reparative responses to injury.^[3–6]^ Recent single-cell RNA-sequencing analyses have deconstructed the arachnoid into 3 functionally distinct strata: an inner fibroblast layer that is directly bathed by cerebrospinal fluid, an intermediate layer of arachnoid barrier cells, and an outer dural border cell layer. These 3-layered organizational principles were subsequently corroborated by immunoconfocal microscopy, immuno-electron microscopy, and analyses of transgenic reporter mouse lines.^[7]^ An independent study has further confirmed the presence of fibroblasts within the arachnoid layer.^[8]^ Fibroblasts exhibit pronounced functional plasticity and lineage flexibility, permitting context-dependent specification toward diverse phenotypes. Investigations of trauma-induced heterotopic ossification have delineated resident connective-tissue progenitors in which injury-responsive signaling suppresses the adipogenic competence of fibroblast-like cells, thereby redirecting differentiation toward chondrogenesis and initiating an osteogenic cascade.^[9,10]^ Moreover, following traumatic brain injury, arachnoid fibroblasts reexpress developmental markers and actively participate in the reparative response.^[7]^ Adhesive arachnoiditis is widely acknowledged as the immediate antecedent to definitive AO. Recent single-cell analyses have identified a distinct fibroblast subpopulation – designated subcluster 1 – within inflamed arachnoidal tissue that displays an osteogenic transcriptional program characterized by up-regulation of BMP, JUNB, SFRP2, and ALPL, all of which are intimately implicated in skeletal development and calcification. These findings furnish molecular evidence elucidating the pathogenesis of AO.^[11]^

In a retrospective case series of 41 patients with confirmed AO, 78% (32/41) had previously undergone spinal surgery – predominantly decompression or fusion procedures – and 87% (36/41) exhibited ossification at the lumbar level, with AO manifesting at a mean interval of 13.4 years after the index operation.^[12]^ In contrast, the index case exhibited markedly abbreviated latency (initial detection at 10 months postoperatively) and was characterized by more extensive ossification, including intrathecal bone formation. This observation further broadens the epidemiological spectrum of AO. Furthermore, based on the 2 AO classification systems reported in the literature, the present case is classified as circumferential ossification encircling the spinal cord, consistent with prior descriptions of progressive neurological worsening.^[5]^ According to an alternative classification system, the case corresponds to Type III, defined by a honeycomb pattern localized to the lumbar spine and cauda equina, with calcifications traversing the thecal sac and potentially ensheathing individual nerve roots.^[13]^ Compared with Types I and II, which predominantly involve the thoracic spine and are frequently complicated by severe canal stenosis and resultant paraplegia, Type III ossification is typically confined to the lumbar spine and is clinically characterized by an exceptionally low incidence of severe or rapidly progressive neurological deficits. These cases corroborated and refined the validity of both classification systems.

According to the first classification system, focal ossified plaques respond favorably to surgical excision, whereas circumferential ossification yields unpredictable postoperative outcomes. Under the alternative scheme, Type III lesions – characterized by limited neural involvement – are generally managed conservatively.^[5,13]^ Surgical intervention is a known etiological factor for AO, making the decision to proceed with surgery controversial. For compressive lesions, the success rates of decompression laminectomy, dural plasty and plaque removal vary.^[14,15]^ However, it is generally advised to avoid attempting the removal of calcified plaques from the spinal cord or nerve roots. This recommendation stems from the fact that calcifications frequently involve and encapsulate the spinal cord or nerve roots, making complete resection challenging. Surgical manipulation in such cases carries a significant risk of exacerbating neurological deficits.^[2]^ Long-term follow-up studies of patients undergoing surgical treatment for AO reveal that improvement is achieved in only half of the cases.^[16,17]^ Although the present patient exhibited extensive ossification on imaging, the therapeutic decision was guided by the literature-based classification criteria, the patient’s relatively mild symptomatology, and the anticipated technical challenges of surgery. Consequently, a conservative approach was adopted; however, the potential need for surgical intervention will be reassessed should disease progression become evident. In recent years, acupuncture has garnered increasing attention from researchers. Some clinical evidence has indicated its advantages in alleviating pain; for example, patients with recurrent lumbar disc herniation often prefer acupuncture and other complementary and alternative medicine (CAM) interventions.^[18–20]^ The mechanism may involve acupuncture-mediated enhancement of peripheral circulation and improvement of local neurovascular perfusion, thereby promoting nerve regeneration.^[21]^ In the present case, the combination of conventional conservative pharmacotherapy with acupuncture as a CAM modality yielded symptomatic relief. These findings suggest that, when surgical indications are absent or operative conditions are unsuitable, conservative pharmacological management together with CAM therapies constitutes a viable therapeutic option.

Several limitations of the present study should be acknowledged. First, the relatively brief posttreatment follow-up interval precludes reliable evaluation of long-term symptomatic improvement and disease progression in AO. Second, pragmatic clinical constraints precluded the application of advanced imaging modalities and histopathological verification, thereby limiting diagnostic precision.

## 4. Conclusion

Our study provides longitudinal radiographic evidence of postoperative progression to AO following lumbar surgery. The patient reported substantial symptomatic improvement after conservative management, despite persistent intrathecal calcification. These findings suggest that there is a viable option for patients with Type III (more severe) AO who exhibit only mild clinical symptoms.

## Author contributions

**Conceptualization:** Xin Xin, Ze-Lin Yue.

**Data curation:** Tong-Hao Sheng, Chuang-Ye Zhang.

**Formal analysis:** Xin Xin, Ze-Lin Yue.

**Funding acquisition:** Xin Xin, Ze-Lin Yue, Chuan-Fu Wei, Nian-Hu Li.

**Investigation:** Xin Xin, Ze-Lin Yue, Tong-Hao Sheng, Chuang-Ye Zhang, Chuan-Fu Wei.

**Methodology:** Xin Xin, Ze-Lin Yue, Jing-Guo Wu.

**Project administration:** Ze-Lin Yue, Jing-Guo Wu.

**Resources:** Chuan-Fu Wei, Nian-Hu Li.

**Supervision:** Jing-Guo Wu, Nian-Hu Li.

**Validation:** Tong-Hao Sheng, Chuang-Ye Zhang, Jing-Guo Wu.

**Writing – original draft:** Xin Xin, Ze-Lin Yue, Chuan-Fu Wei.

**Writing – review & editing:** Jing-Guo Wu, Nian-Hu Li.
